# Effects of indulgent food snacking, with and without exercise training, on body weight, fat mass, and cardiometabolic risk markers in overweight and obese men

**DOI:** 10.14814/phy2.15118

**Published:** 2021-11-24

**Authors:** Wesley J. Tucker, Catherine L. Jarrett, Andrew C. D’Lugos, Siddhartha S. Angadi, Glenn A. Gaesser

**Affiliations:** ^1^ College of Health Solutions Arizona State University Phoenix Arizona USA

**Keywords:** body fat, donuts, endothelial function, energy compensation, obesity, sugar

## Abstract

We hypothesized that exercise training would prevent gains in body weight and body fat, and worsening of cardiometabolic risk markers, during a 4‐week period of indulgent food snacking in overweight/obese men. Twenty‐eight physically inactive men (ages 19–47 yr) with body mass index (BMI) ≥25 kg/m^2^ consumed 48 donuts (2/day, 6 days/week; ~14,500 kcal total) for 4 weeks while maintaining habitual diet. Men were randomly assigned to control (*n* = 9), moderate‐intensity continuous training (MICT; *n* = 9), or high‐intensity interval training (HIIT; *n* = 10). Exercise training occurred 4 days/week, ~250 kcal/session. Controls did not increase body weight, body fat, or visceral abdominal fat. This was partially explained by a decrease in self‐reported habitual energy (−239 kcal/day, *p *= 0.05) and carbohydrate (−47 g/day; *p* = 0.02) intake. Large inter‐individual variability in changes in body weight, fat, and fat‐free mass was evident in all groups. Fasting blood pressure, and blood concentrations of glucose, insulin, and lipids were unchanged in all groups. Glucose incremental area under the curve during an oral glucose tolerance test was reduced by 25.6% in control (*p* = 0.001) and 32.8% in MICT (*p* = 0.01) groups. Flow‐mediated dilation (FMD) was not changed in any group. VO_2max_ increased (*p* ≤ 0.001) in MICT (9.2%) and HIIT (12.1%) groups. We conclude that in physically inactive men with BMI ≥25 kg/m^2^, consuming ~14,500 kcal as donuts over 4 weeks did not adversely affect body weight and body fat, or several markers of cardiometabolic risk. Consumption of the donuts may have prevented the expected improvement in FMD with HIIT.

## Graphical abstract

Despite ingesting 48 donuts (~14,500 kcal) over 4 weeks, overweight and obese men did not gain weight or body fat, and actually experienced an increase in oral glucose tolerance. HIIT and MICT improved cardiorespiratory fitness during the 4 weeks of indulgent food snacking but did not improve most cardiometabolic risk markers. Consumption of the donuts may have prevented expected improvements in endothelial function following HIIT.

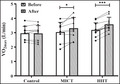

## 1 INTRODUCTION

Snacking has increased during the past 50 years and may contribute to excess energy intake and adversely affect diet quality (Bellisle, 2014; Mattes, 2018). Thus, snacking may influence energy balance and markers of cardiometabolic health. Snack foods can vary considerably in nutrient and energy density, which can alter the impact of the snack on energy balance and health outcomes. Randomized controlled trials of snacking have shown that snacks such as fruits, vegetables, nuts, and cereal bars have little, if any, effect on body weight, body fat, or cardiometabolic risk markers (Claesson et al., 2009; Njike et al., 2017; Viskaal‐van Dongen et al., 2010; Zaveri & Drummond, 2009). By contrast, indulgent snack foods consisting mostly of fat and sugar have been shown to increase body weight (Claesson et al., 2009; Mazlan et al., 2006; Njike et al., 2017), blood pressure (Njike et al., 2017), and fasting insulin concentration (Claesson et al., 2009). Positive energy balance in these studies may be due to the fact that the mandatory snacks were cookies and candy, which are ultra‐processed foods that have been associated with increased overall dietary energy intake and weight gain (Hall et al., 2019).

An important question is whether exercise can mitigate the deleterious effects of mandatory consumption of indulgent snack foods. In young men, exercise training has been reported to prevent weight gain during 1 week of consuming 900 kcal/day of sugar‐sweetened beverages while otherwise maintaining normal dietary intake (Bock et al., 2020), and during 14 days of consuming fast‐food meals (three extra value meals/day + optional snack) as the sole source of energy intake (Duval et al., 2017). Also, a high level of physical activity (~11,000–12,500 steps/day) has been shown to offset the deleterious effects of 14 days of a high‐fructose diet (Bidwell et al., 2014) or excess energy intake (Krogh‐Madsen et al., 2014). However, these studies used subjects with a mean body mass index (BMI) <25 kg/m^2^. In fact, most of the studies on snacking used subjects with BMI <30 kg/m^2^ (Mazlan et al., 2006; Tey et al., 2011; Viskaal‐van Dongen et al., 2010). In two studies that recruited only participants with BMI ≥25 kg/m^2^ (Njike et al., 2017; Zaveri & Drummond, 2009), exercise was not included in the research design. Only one of these studies examined snack food options comprised of mostly fat and sugar (cookies), and the investigators reported significant increases in body weight, percent body fat, and systolic and diastolic blood pressures (Njike et al., 2017).

Thus, the primary purpose of our study was to determine whether exercise training could offset the expected increases in body weight and body fat resulting from consumption of an indulgent snack food 6 days per week for 4 weeks in physically inactive men with a BMI ≥25 kg/m^2^. Secondarily, we were interested in determining whether several indices of cardiometabolic health would be adversely affected by the indulgent snack food consumption, and if exercise training could prevent these from occurring. We chose donuts as our indulgent snack because they are highly palatable, consist of mostly fat and sugar, and are widely consumed (~10 billion donuts are consumed annually in the United States (DMR Business Statistics, 2021). We used both high‐intensity interval training (HIIT) and moderate‐intensity continuous training (MICT) in order to compare the effectiveness of each type of training on mitigating the anticipated adverse effects of the donuts. We chose a 4‐week intervention because this duration may have relevance for interpreting results of studies on holiday weight gain (Cook et al., 2012; Ramirez‐Jimenez et al., 2020; Stevenson et al., 2013; Yanovski et al., 2000). For example, it was recently reported that during the Christmas holiday period, 3 weeks of HIIT prevented weight gain and increases in blood pressure, fasting insulin concentration, and HOMA‐IR in overweight and obese men with metabolic syndrome (Ramirez‐Jimenez et al., 2020).

We hypothesized that both exercise training protocols would result in significantly less gain in body weight and fat mass during the 4‐week period of indulgent food snacking compared to a sedentary, control group, with no differences between exercise training groups. Based on limited published data, we also hypothesized that both exercise protocols would prevent expected increases in blood pressure (Njike et al., 2017) and fasting insulin concentration (Claesson et al., 2009). We also included measurements of glucose tolerance, blood lipids, endothelial function, and cardiorespiratory fitness as additional outcomes.

## 2 MATERIALS AND METHODS

### 2.1 Subjects

This study was approved by the Arizona State University Institutional Review Board (IRB #00001517) and conformed to the ethical standards of the Declaration of Helsinki. All subjects provided informed written consent before participation. Inclusion criteria were as follows: men ages 18–50, nonsmoking, BMI between 25 and 45 kg/m^2^, currently physically inactive (Tremblay et al., 2017) but capable of performing vigorous physical activity, providing “no” answers to any question on the Physical Activity Readiness Questionnaire (PAR‐Q) (Thomas et al., 1992), not currently taking any dietary supplements or medications for cardiovascular disease, hypertension or diabetes, and a willingness to consume two donuts per day, 6 days per week for 4 weeks. Thirty‐three men were screened, and 28 met all inclusion criteria and were enrolled in the study (Table 1).

**TABLE 1:** Baseline subject characteristics

	Control (*n* = 9)	MICT (*n* = 9)	HIIT (*n* = 10)
Age (years)	28 ± 9	29 ± 7	30 ± 7
Height (cm)	177.8 ± 7.5	178.6 ± 10.1	178.1 ± 5.5
Weight (kg)	93.1 ± 11.8	94.5 ± 18.6	96.0 ± 7.7
Body Mass Index (kg/m²)	29.6 ± 3.9	29.7 ± 4.5	30.2 ± 3.0
Body Fat Percentage (%)	31.5 ± 5.9	31.1 ± 5.6	30.5 ± 4.5
Fat Mass (kg)	29.5 ± 8.5	26.7 ± 7.5	30.4 ± 9.1
Fat‐Free Mass (kg)	64.2 ± 5.9	63.8 ± 9.6	68.7 ± 6.1
VO_2max_ (L/min)	3.0 ± 0.5	3.1 ± 0.7	3.2 ± 0.5
VO_2max_ (ml/kg/min)	31.4 ± 4.1	33.5 ± 4.9	33.2 ± 6.9

Note

Data represent mean ± SD. No group differences present.

HIIT, High‐intensity interval training; MICT, Moderate‐intensity continuous training; VO_2max_, Maximal oxygen uptake.

### 2.2 Study design

A randomized, parallel‐group design was used for this study. Following baseline assessments, participants were randomly assigned to one of three different groups: donut snacking without exercise training (Control, *n* = 9), donut snacking plus 1,000 kcal/week of exercise as moderate‐intensity continuous training (MICT, *n* = 9), and donut snacking plus 1,000 kcal/week of exercise as high‐intensity interval training (HIIT, *n* = 10) for 4 weeks. Body weight and fat mass were the primary outcomes for this study. Secondary outcomes included blood pressure, glycemic control, blood lipids, endothelial function, and cardiorespiratory fitness.

All experimental testing took place in a climate‐controlled research laboratory. All subjects reported to the laboratory for baseline testing in the morning following a ≥10 h overnight fast. Subjects were instructed to refrain from exercise, caffeine, and alcohol for ≥48 h prior to each testing visit. Body composition, blood pressure, endothelial function, glycemic control, and cardiorespiratory fitness were all assessed in that order at each testing visit. For MICT and HIIT, post‐testing was carried out ≥48 h after the last exercise training session to avoid the acute effects of the last exercise bout (Mikines et al., 1989; Sylow et al., 2017). Control group subjects were assessed ≥48 h after consuming their last donut. For each subject, the order and time of day for conducting these testing procedures were held constant to reduce the impact of diurnal variations on physiological outcome measures.

### 2.3 Anthropometrics, body composition, and energy balance

Height and weight were assessed with a digital stadiometer (Seca 284, Seca, Hamburg, Germany). Dual‐energy X‐ray Absorptiometry (DXA) (Lunar iDXA, GE Healthcare, Little Chalfont, UK) was used to determine total percent body fat (%), fat mass (kg), and fat‐free mass (kg), as well as regional fat distributions and visceral fat. Changes in energy balance for each subject during the 4‐week intervention were estimated using the following formula: Energy balance = (Δ lean mass [kg] × 1,100 kcal/kg) + (Δ fat mass [kg] × 9,540 kcal/kg) (King et al., 2009; Sawyer et al., 2015a).

### 2.4 Blood pressure

Brachial artery and central aortic blood pressures were assessed using the SphygmoCor XCEL device (AtCor Medical Pty, West Ryde, NSW, Australia) (Gallagher et al., 2004; Karamanoglu et al., 1993). All measurements were performed in accordance with manufacturer recommendations. Subjects rested in a supine position in a quiet, dimly lit, climate‐controlled room for a minimum of 15 min prior to blood pressure measurements. Thereafter, brachial systolic blood pressure and diastolic blood pressure were measured at the brachial artery in the left arm with an appropriately sized blood pressure cuff. At the same time, brachial artery waveforms were recorded for 5 seconds and used to generate central aortic waveforms using a validated transfer function (Gallagher et al., 2004). This allows for the estimation of central systolic and diastolic blood pressures, which provide estimates of the maximum and minimum pressures in the aorta, respectively. All blood pressure measurements were done in triplicate, with the two closest values being averaged and used.

### 2.5 Endothelial function

Endothelial function was assessed by flow‐mediated dilation (FMD) of the brachial artery with a high‐resolution ultrasound system (Terason t3000CV Ultrasound, Terason, Burlington, MA) following the latest established guidelines for the assessment of FMD (Harris et al., 2010; Thijssen et al., 2011, 2019). This ultrasound system had a 12‐MHz linear array probe with 60 degrees of insonation. The same well‐trained sonographer performed all FMDs. Measurements were made in a quiet, dimly lit, climate‐controlled room after 20 min of supine rest following confirmation of hemodynamic stability. All brachial artery images were taken on the subject's left arm (immobilized) in the longitudinal plane, proximal to antecubital fossa. The ultrasound procedure was individualized to optimize image clarity and avoid vessel branching. Probe location and image settings for the baseline assessment were recorded and repeated for the post‐intervention assessment to ensure high reproducibility within‐subject. A 1‐min, B‐mode video was captured to determine resting baseline brachial artery diameter. Thereafter, a blood pressure cuff (Hokanson E20 Rapid Cuff Inflator, Hokanson Inc., Bellevue, WA, USA) was placed 3 cm distal to the antecubital fossa and rapidly inflated to >200 mmHg (or greater than 50 mmHg above systolic blood pressure) for 5 min. Digital video was recorded for 1 min prior to cuff release and for a minimum of 3 min following cuff release to assess peak artery diameter, shear rate, and average blood velocity. Arterial diameters were calculated as the distance between anterior and posterior walls of the intima‐lumen surface.

FMD was defined as the change in arterial diameter from resting baseline to peak dilation following cuff release as a percentage of baseline diameter. Ultrasound images were recorded at 30 frames/second using Camtasia software (TechSmith, Okemos, MI, USA). All ultrasound images were analyzed in duplicate using semi‐automated edge‐detection software (Woodman et al., 2001) by a single investigator who was blinded to both time and group allocation. Intra‐user reliability was as follows: baseline diameter (CV% = 0.2%, ICC = 0.99), peak diameter (CV% = 0.2%, ICC = 0.99), and FMD% (CV% = 4.2%, ICC = 0.97). A second investigator, also blinded to the experimental conditions, analyzed a subset of the images (n = 40) to assess inter‐observer reliability. Inter‐observer reliability was as follows: baseline diameter (CV% = 0.9%, ICC = 0.98), peak diameter (CV% = 1.6%, ICC = 0.96), and FMD% (CV% = 8.2%, ICC = 0.97).

### 2.6 Oral glucose tolerance testing and blood analyses

Oral glucose tolerance tests (OGTTs) were performed after an overnight fast. An IV catheter was placed into an antecubital vein and venous blood samples were collected at baseline and every 30 min (−30, 0, 30, 60, 90, 120 min; 75 g anhydrous glucose was ingested at timepoint 0). After each blood draw during the OGTT, the blood was placed into appropriately labeled vacutainers, processed, and stored at −80°C until assayed. Serum glucose concentration was analyzed using an automated chemistry analyzer (Cobas C111, Roche Diagnostics, Indianapolis, IN) using colorimetric enzymatic agents. Serum insulin concentration was measured using the ultrasensitive human radioimmunoassay kit (Millipore Corporation, Billerica, MA).

Fasting serum lipids (total cholesterol, high‐density lipoprotein cholesterol (HDL‐c), low‐density lipoprotein cholesterol (LDL‐c), and triglycerides) were analyzed using an automated chemistry analyzer (Cobas C111, Roche Diagnostics, Indianapolis, IN) using colorimetric enzymatic agents. All samples were run in duplicates and the mean value was calculated. Measured intra‐assay CVs were as follows: glucose = 0.46%, insulin = 4.9%, total cholesterol = 0.46%, HDL‐c = 0.45%, LDL‐c = 0.52%, and triglycerides = 0.79%.

### 2.7 Cardiorespiratory fitness

Maximum oxygen uptake (VO_2max_) was assessed during an incremental ramp test on an electronically braked cycle ergometer (VIAsprint, 150P, Ergoline, Bitz, Germany). Pulmonary ventilation and gas exchange were continuously measured via indirect calorimetry with a Parvo Medics TrueOne 2400 (Parvo Medics, Sandy, UT, USA) computerized metabolic measurement system. Heart rate was continuously measured with a Polar heart rate monitor (Polar Electro Inc., Lake Success, NY). A standard three‐point calibration was performed prior to each test per manufacturer recommendations. After 2 min of seated rest, subjects pedaled on a cycle ergometer at a cadence of their choice (60–80 rpm) at 50 W for 5 min for the warm‐up phase. After the warmup, power increased continuously in a ramp fashion (1 W every 2 s) until each subject reached volitional exhaustion despite strong verbal encouragement. After a 10‐min cool‐down phase of cycling at 50 W, each subject performed a verification phase test consisting of cycling at a constant power output equivalent to 100% of maximum power achieved on the incremental ramp test (Sawyer et al., 2015b; Tucker et al., 2015b). Subjects were asked to keep their cadence above 50 rpm and pedal for as long as possible during the verification phase. VO_2max_ was defined as the mean of the two highest consecutive 15‐sec averages attained for VO_2_ during either the incremental ramp or verification phase of the maximal exercise test. Maximal heart rate (HR_max_) was defined as the highest HR achieved during either the incremental ramp or verification phase.

### 2.8 Habitual dietary intake and physical activity levels

All subjects were asked to keep track of their habitual dietary intake by filling out a Three‐Day Food Record at baseline (pre‐intervention) and during week 4 of the intervention. Dietary intake was recorded on 2 weekdays and 1 weekend day and analyzed with nutritional analysis software (Diet Analysis Plus 10.0, Cengage, Independence, KY, USA) by a registered dietitian.

To assess habitual physical activity levels, all subjects wore a validated physical activity monitor (SenseWear Armband, Model: WMS, Bodymedia, Pittsburgh, PA, USA) (Bhammar et al., 2016; Jakicic et al., 2004; Johannsen et al., 2010; Tucker et al., 2015a) for 7 days prior to baseline testing and again during week 4 of the intervention. Daily steps, and number of daily min of sedentary behavior and moderate‐to‐vigorous physical activity (MVPA) were measured and recorded during each day of wear. Subjects were instructed to wear the armband on the upper posterior aspect of the left arm throughout the day but permitted to remove the armband at night to avoid discomfort during sleep. In addition, the armband was removed prior to each exercise training session in subjects randomized to the exercise training groups.

### 2.9 Indulgent snack food

All subjects were instructed to maintain their current diet while also consuming two donuts per day, 6 days per week, for 4 weeks. Donuts were purchased by our research team at a local Dunkin Donuts (Dunkin’ Brands, Canton, MA, USA) and given to our subjects to consume as snacks between meals. Subjects picked up the donuts from our laboratory three times per week in packages of four donuts (for freshness). For variety, subjects were allowed to select from a menu of donut options, in which donuts were categorized by energy content. By requiring subjects to select donuts in equal proportions from the different categories, this ensured that the total energy content of the donuts would be similar across subjects in the three groups. Donut nutrition information was obtained from the Dunkin Donuts website. All donuts contained between 250 and 350 kcal, of which at least 70% of total calories were fat and sugar. Energy content of the two donuts consumed daily was ~600 kcal, with the cumulative total energy value of ~14,500 kcal during the 4 weeks. A full energy and macronutrient profile of the 48 donuts consumed during the 4‐week intervention is provided in Table 2.

**TABLE 2:** Donut nutritional information

	Control (*n* = 9)	MICT (*n* = 9)	HIIT (*n* = 10)
Donuts			
Total energy (kcal)	14,554 ± 572	14,654 ± 204	14,542 ± 398
Carbohydrate (g)	1,686 ± 61	1,704 ± 50	1,688 ± 54
Fat (g)	815 ± 41	808 ± 19	805 ± 39
Protein (g)	153 ± 5	157 ± 6	154 ± 3
Sugar (g)	731 ± 42	749 ± 18	739 ± 34
Saturated fat (g)	360 ± 22	355 ± 10	355 ± 17
Fiber (g)	50 ± 3	53 ± 5	52 ± 3
Macronutrient distribution			
Carbohydrate (%)	46 ± 1	46 ± 1	46 ± 1
Fat (%)	50 ± 1	50 ± 1	50 ± 1
Protein (%)	4 ± 0	4 ± 1	4 ± 0

Note

Data represent mean ± SD for the total 48 donuts consumed during the 4‐week snacking intervention. No group differences present.

Abbreviations: HIIT, High‐intensity interval training; MICT, Moderate‐intensity continuous training.

### 2.10 Exercise training

All subjects randomized to exercise training (MICT or HIIT) completed four supervised exercise training sessions per week for 4 weeks, for a total of 16 exercise sessions. All exercise training was performed on mechanically braked cycle ergometers (Monark Ergomedic 828E, Monark, Sweden). Heart rate was continuously monitored using a Polar heart rate monitor (Polar H7, Polar Electro OY, Kempele, Finland) and recorded during all exercise training sessions. MICT sessions consisted of a 5‐min warm‐up (50 W), 30–45 min of cycling at a HR associated with 50% VO_2max_, and a 5‐min cool down (50 W). HIIT sessions consisted of a 5‐min warm‐up (50 W), 8–11, 1‐min cycling intervals at 90–95% HR_max_ interspersed by 1‐min active recovery periods (50 W), and a 5‐min cool down (50 W). To ensure that exercise protocols were isoenergetic, total exercise duration was individualized based on baseline VO_2max_ to achieve approximately 250 kcal total energy expenditure per session, including warm‐up and cool‐down.

Subjects randomized to the control group were instructed to maintain their current physical activity pattern for the duration of the study. As incentive, control group subjects were offered 4 weeks of free exercise training (either MICT or HIIT) upon completion of the study.

### 2.11 OGTT calculations

Total area under the curve (tAUC) and incremental area under the curve (iAUC) for glucose and insulin, and OGTT‐derived indices of insulin action were used to assess changes in glycemic control during the intervention. The trapezoidal rule was used to calculate tAUC by subtracting zero from the AUC and iAUC for glucose and insulin by subtracting the fasting value from AUC (Wolever & Jenkins, 1986). Insulin sensitivity index (ISI) was estimated by Matsuda Index (Matsuda & DeFronzo, 1999). The product of the glucose and insulin tAUCs during the first 30 min of the OGTT was used to calculate the hepatic insulin resistance index (IRI) (Abdul‐Ghani et al., 2007) as follows: IRI = (glucose tAUC_0‐30min_ × insulin tAUC_0‐30min_). Hepatic IRI was divided by 1,000 for data presentation. The early insulin response (insulinogenic index [IGI]) was calculated as the ratio of the change in insulin concentration to the change in glucose concentration during the first 30 min of OGTT (Abdul‐Ghani et al., 2007) as follows: IGI = (insulin iAUC_0‐30min_ /glucose iAUC_0‐30min_). Beta‐cell function was estimated using the oral disposition index (DI) as follows: DI = IGI X Matsuda ISI (Abdul‐Ghani et al., 2007). The homeostatic model of assessment (HOMA‐IR) score was calculated as follows: HOMA‐IR = glucose (mmol/L) × insulin (μU/ml)/22.5).

### 2.12 FMD data analysis

Inadequate scaling for FMD was not present as evidenced by the upper confidence limit of the regression slope of the relationship between the logarithmically transformed baseline and peak brachial artery diameters exceeding 1 (β ± SE = 0.94; 95% CI = 0.86 to 1.02) (Atkinson et al., 2013). Therefore in accordance with the guidelines outlined by Atkinson et al. (Atkinson et al., 2013), allometric scaling was deemed unnecessary and the % FMD variable was utilized as the dependent variable.

### 2.13 Sample size estimates and statistical analysis

Based on unpublished pilot data from our laboratory (Black, 2013), we calculated that the completion of 30 participants would yield 90% power to detect a 1.9 kg difference in body weight between the control and exercise conditions (two‐tailed α = 0.05) using G*Power 3.1 software (Faul et al., 2007).

All statistical analyses were performed with SPSS (SPSS 24.0, IBM, Armonk, NY) and Prism Software (GraphPad version 8, La Jolla, CA). All *p* values were two‐sided and α < 0.05 was considered statistically significant. All variables are presented as mean ± SD, unless stated otherwise. All variables were checked regarding normal distribution using the Shapiro–Wilk test and logarithmically transformed if appropriate. Two‐way repeated measures analysis of variance (ANOVA) was used to assess the effect of time, condition, and time x condition interaction for all physiological variables studied. If the sphericity assumption was violated (Greenhouse‐Geisser ε < 0.75), degrees of freedom (df values) for within‐subject were adjusted using Greenhouse‐Geisser correction. The Bonferroni adjustment was used for multiple comparisons when appropriate. One‐way ANOVAs were used to assess group differences in subject characteristics at baseline, dietary composition of the donuts, and changes in calculated energy balance based on DXA results.

## 3 RESULTS

### 3.1 Habitual physical activity and dietary intake

There was no change in objectively measured habitual physical activity in any group during the 4‐week intervention (Table 3). Not including consumption of the donuts, habitual daily carbohydrate intake was lower by ~47 g (*p* = 0.02) and total energy intake lower by ~239 kcal (*p* = 0.05) in the control group, when comparing baseline and self‐reported dietary intake during the fourth week of the study (Table 3). Habitual dietary intake was unchanged in both MICT and HIIT groups.

**TABLE 3:** Habitual physical activity levels and dietary intake

	Control (*n* = 9)			MICT (*n* = 8)			HIIT (*n* = 10)			
	Before	After	*p*	Before	After	*p*	Before	After	*p*	*Inter p*
Habitual physical activity										
Steps per day	6,417 ± 1,435	6,352 ± 1,584	0.92	7,038 ± 2,769	7,317 ± 2,861	0.68	6,366 ± 2,111	6,556 ± 2,759	0.75	0.93
Sedentary (min/day)	579 ± 56	569 ± 60	0.58	533 ± 50	511 ± 86	0.25	585 ± 61	583 ± 58	0.89	0.74
MVPA (min/day)	94 ± 55	97 ± 49	0.80	121 ± 79	129 ± 84	0.41	89 ± 29	83 ± 32	0.55	0.59
Habitual dietary intake										
TEI (kcal/day)	2,406 ± 486	2,167 ± 560	0.05	2,483 ± 563	2,445 ± 447	0.77	2,426 ± 381	2,383 ± 366	0.71	0.42
Carbohydrate (g)	283 ± 65	236 ± 80	0.02*	319 ± 107	306 ± 97	0.54	260 ± 47	244 ± 44	0.39	0.41
Fat (g)	93 ± 21	87 ± 20	0.39	95 ± 21	93 ± 22	0.82	98 ± 23	101 ± 20	0.76	0.71
Protein (g)	100 ± 20	100 ± 24	0.96	99 ± 37	92 ± 30	0.51	106 ± 16	112 ± 41	0.53	0.66

Note

Data represent mean ± SD.

Abbreviations: HIIT, High‐intensity interval training; Inter *p*, Interaction *p*‐value; MICT, Moderate‐intensity continuous training; MVPA, Moderate‐to‐vigorous physical activity; TEI, Total energy intake.

* denotes significant difference *p* < 0.05 within condition.

### 3.2 Body composition and energy balance

**FIGURE 1:**
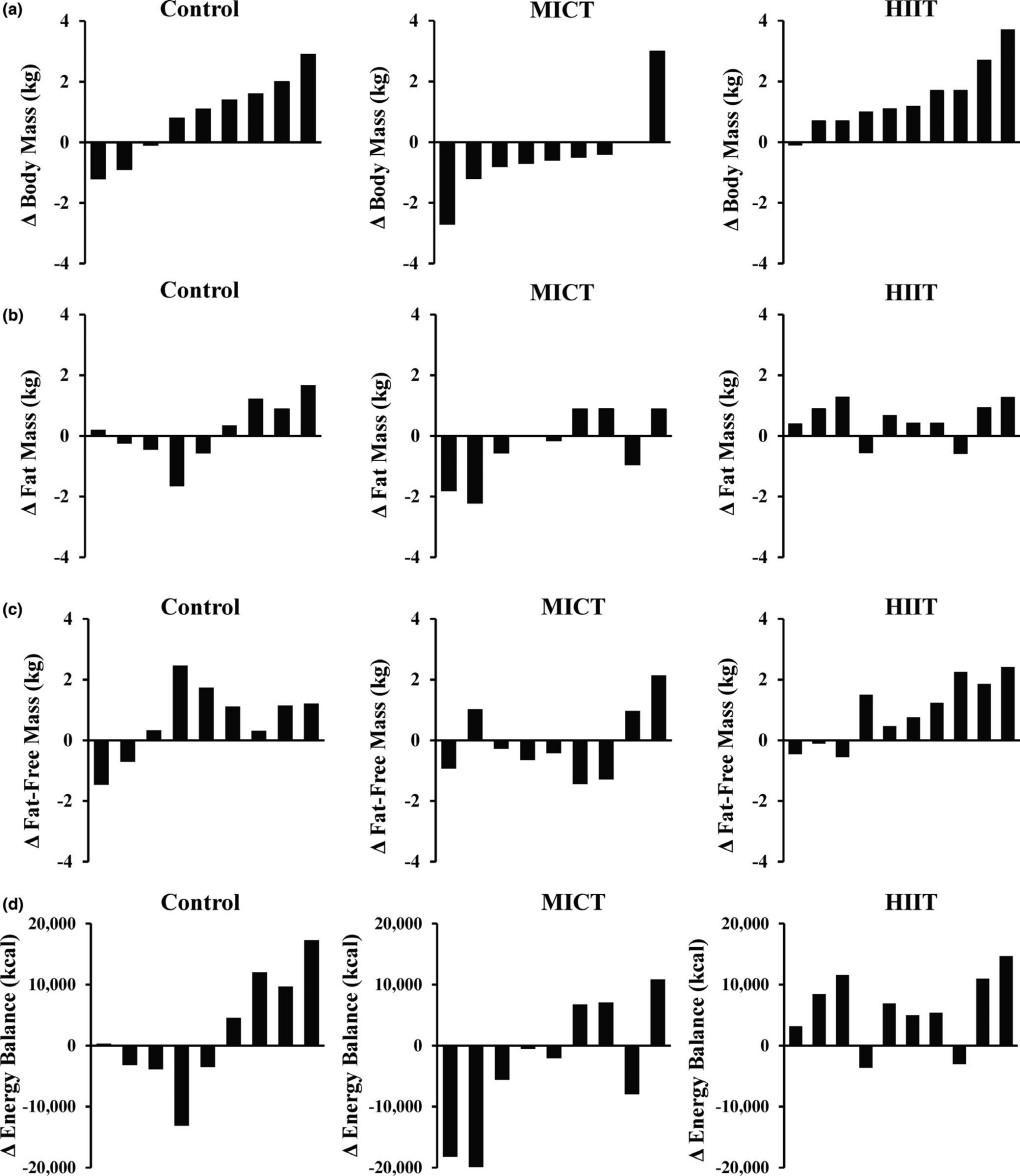
Individual changes in body mass (kg) (panel A), fat mass (kg) (panel B), fat‐free mass (kg) (panel C), and energy content (kcal) (panel D) following the 4‐week snacking intervention for control, moderate‐intensity continuous training (MICT), and high‐intensity interval training (HIIT) groups

Changes in total and regional body composition are presented in Table 4. There was a time x group interaction for body weight (*p* = 0.02), with a mean increase in HIIT (+1.2 kg; *p* = 0.02) and no change in both control and MICT. The increase in body weight in HIIT was primarily due to a mean increase of 0.9 kg for fat‐free mass (*p* = 0.02). Fat mass was unchanged in all groups. Changes in energy balance from the DXA revealed no differences between groups (Control: +2,228 ± 9,494 kcal, MICT: −3,291 ± 10,582 kcal, HIIT: +5,942 ± 5,969 kcal) (*p* = 0.10). Despite the relatively unchanged mean data for body composition, there was considerable inter‐individual variability for changes in body weight (−1.8 kg to +3.1 kg) (Figure 1A), fat mass (−2.2 kg to +1.7 kg) (Figure 1B), fat‐free mass (−1.5 kg to +2.5 kg) (Figure 1C), and body energy content based on DXA (−19,884 kcal to +17,241 kcal) (Figure 1D) across all groups during the 4‐week intervention.

**TABLE 4:** Total and regional body composition

	Control (*n* = 9)			MICT (*n* = 9)				HIIT (*n *= 10)			
	Before	After	*p*		Before	After	*p*	Before	After	*p*	Inter *p*
Total body composition											
Body mass (kg)	93.1 ± 11.8	94.0 ± 12.8	0.07		94.5 ± 18.6	94.1 ± 17.9	0.33	96.0 ± 7.7	97.4 ± 7.9	0.02*	0.02*
Fat mass (kg)	28.7 ± 8.7	28.9 ± 8.9	0.63		29.8 ± 11.0	29.5 ± 10.9	0.31	28.3 ± 5.3	28.9 ± 5.3	0.10	0.18
Fat‐free mass (kg)	64.4 ± 6.1	65.1 ± 6.7	0.09		64.7 ± 10.7	64.6 ± 9.7	0.81	67.7 ± 4.8	68.6 ± 4.6	0.02*	0.1
Regional adiposity											
Android fat mass (%)	39.6 ± 9.8	39.4 ± 9.6	0.66		39.6 ± 11.7	39.4 ± 11.4	0.57	39.2 ± 6.3	39.6 ± 6.1	0.41	0.55
Gynoid fat mass (%)	30.7 ± 4.3	30.8 ± 4.3	0.84		31.8 ± 5.3	31.5 ± 5.0	0.44	29.2 ± 4.3	28.7 ± 3.6	0.13	0.49
Visceral adipose tissue (cm^3^)	1,384 ± 1,246	1,382 ± 1,175	0.97		1,278 ± 1,015	1,271 ± 1,002	0.88	1,385 ± 467	1,473 ± 564	0.04*	0.21

Note

Data represent mean ± SD.

Abbreviations: HIIT, High‐intensity interval training; Inter *p*, Interaction *p*‐value; MICT, Moderate‐intensity continuous training.

* denotes significant difference *p* < 0.05.

Android and gynoid fat mass percentages were unchanged across groups (Table 4). However, visceral adipose tissue increased in HIIT by 6.4% (~88 cm^3^; *p* = 0.04) with no changes observed in control and MICT.

### 3.3 Cardiometabolic risk factors

Changes in cardiometabolic risk factors are presented in Table 5. Brachial artery and central aortic blood pressures were unchanged across all groups. Fasting glucose and insulin concentrations were also unchanged after the 4‐week intervention. For the 2‐h OGTT, there was a time × group interaction for glucose iAUC (*p* = 0.04), with reductions in iAUC for both control (*p* = 0.001) and MICT (*p* = 0.01), but no change in the HIIT group. Insulin iAUC, hepatic IRI, IGI, DI, ISI, and HOMA‐IR were unchanged across all groups. Blood lipid variables were also unchanged after the 4‐week intervention.

**TABLE 5:** Cardiometabolic risk factors

	Control (*n* = 9)			MICT (*n* = 8)			HIIT (*n* = 10)			
	Before	After	*p*	Before	After	*p*	Before	After	*p*	Inter *p*
Blood pressures										
Brachial SBP (mmHg)	127 ± 8	128 ± 10	0.55	122 ± 12	123 ± 12	0.69	127 ± 5	125 ± 6	0.58	0.68
Brachial DBP (mmHg)	77 ± 6	75 ± 10	0.47	72 ± 6	73 ± 10	0.68	75 ± 5	76 ± 6	0.69	0.65
Central SBP (mmHg)	113 ± 8	114 ± 9	0.61	108 ± 10	109 ± 11	0.50	113 ± 4	112 ± 5	0.65	0.68
Central DBP (mmHg)	78 ± 6	76 ± 10	0.39	73 ± 6	74 ± 10	0.71	75 ± 4	76 ± 7	0.54	0.53
Glucose & insulin indices										
Fasting glucose (mg/dl)	96 ± 5	94 ± 5	0.35	95 ± 9	94 ± 8	0.51	94 ± 7	94 ± 8	0.72	0.91
Glucose iAUC 0–120 min	5,666 ± 2,826	4,214 ± 2,194	0.001*	3,843 ± 2,274	2,582 ± 2,400	0.01*	3,383 ± 2,154	3,314 ± 1,966	0.87	0.04*
Fasting insulin (µU/ml)	13 ± 5	15 ± 7	0.22	11 ± 4	11 ± 5	0.59	12 ± 4	12 ± 3	0.91	0.32
Insulin iAUC 0–120 min	11,953 ± 7,884	12,083 ± 7,801	0.92	7,876 ± 4,527	6,873 ± 4,526	0.25	7,469 ± 3,282	7,434 ± 3,021	0.97	0.61
Hepatic IRI	8.4 ± 5.2	9.9 ± 7.4	0.11	6.3 ± 3.5	5.0 ± 2.2	0.18	5.9 ± 2.3	5.4 ± 2.3	0.56	0.10
Insulinogenic Index (IGI)	1.8 ± 1.8	2.3 ± 1.6	0.40	2.7 ± 4.3	2.5 ± 2.1	0.73	2.6 ± 3.4	1.9 ± 1.8	0.31	0.41
Disposition Index (DI)	4.8 ± 6.7	6.4 ± 6.1	0.53	7.7 ± 9.2	10.3 ± 7.9	0.36	8.3 ± 10.5	6.4 ± 6.4	0.47	0.46
Matsuda Index (ISI)	2.9 ± 1.8	2.9 ± 1.6	0.91	4.1 ± 2.3	4.5 ± 2.4	0.19	3.7 ± 1.6	3.5 ± 0.7	0.61	0.42
HOMA‐IR	3.2 ± 1.2	3.4 ± 1.8	0.29	2.5 ± 1.0	2.6 ± 1.2	0.73	2.8 ± 1.0	2.6 ± 0.6	0.44	0.43
Blood lipids										
Total cholesterol (mg/dl)	157 ± 14	157 ± 18	0.96	160 ± 29	153 ± 31	0.40	190 ± 27	185 ± 43	0.48	0.77
LDL cholesterol (mg/dl)	103 ± 15	105 ± 19	0.68	108 ± 32	100 ± 30	0.27	135 ± 30	133 ± 43	0.76	0.54
HDL cholesterol (mg/dl)	42 ± 8	44 ± 7	0.49	42 ± 11	44 ± 15	0.40	44 ± 8	44 ± 7	0.68	0.60
Triglycerides (mg/dl)	114 ± 46	113 ± 38	0.93	106 ± 42	111 ± 29	0.74	136 ± 39	117 ± 38	0.11	0.59

Note

Data represent mean ± SD.

Abbreviations: DBP, Diastolic blood pressure; HDL, High‐density lipoprotein; HIIT, High‐intensity interval training; HOMA‐IR, Homeostatic model assessment of insulin resistance; iAUC, Incremental area under the curve; Inter *p*, Interaction *p*‐value; IRI, Insulin resistance index; LDL, Low‐density lipoprotein; MICT, Moderate‐intensity continuous training; SBP, Systolic blood pressure

* denotes significant difference *p* < 0.05.

### 3.4 Endothelial function

**FIGURE 2:**
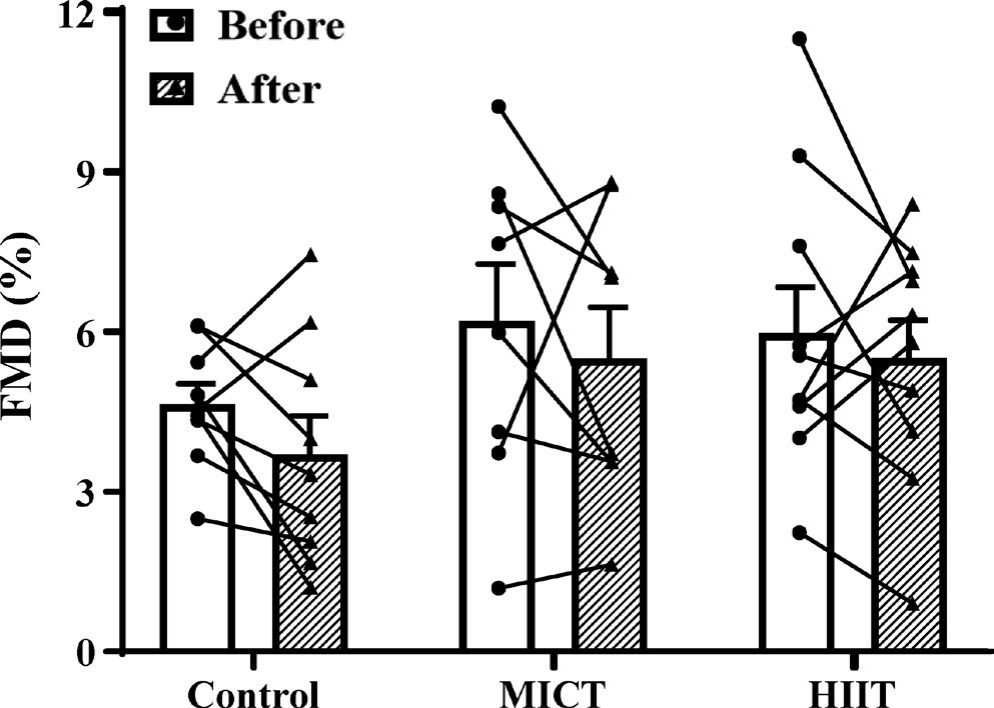
Flow‐mediated dilation (FMD) (%) before (open bars) and after (shaded bars) the 4‐week snacking intervention for control, moderate‐intensity continuous training (MICT), and high‐intensity interval training (HIIT) groups. Filled symbols represent individual data. Group data (bars) presented as means ± SE

Flow‐mediated dilation was unchanged within all groups (*p* > 0.05), and there was no time x group interaction for FMD (*p* = 0.92) (Figure 2). Baseline diameter, peak diameter, peak shear rate, and average blood velocity were unchanged across all groups (Table 6).

**TABLE 6:** Brachial artery ultrasound measurements

	Control (*n* = 9)			MICT (*n* = 8)				HIIT (*n* = 10)			
	Before	After	*p*		Before	After	*p*	Before	After	*p*	Inter *p*
Baseline diameter (mm)	4.2 ± 0.2	4.4 ± 0.2	0.10		4.0 ± 0.2	4.1 ± 0.2	0.53	4.2 ± 0.1	4.3 ± 0.1	0.21	0.27
Peak diameter (mm)	4.4 ± 0.2	4.5 ± 0.2	0.23		4.2 ± 0.2	4.2 ± 0.2	0.59	4.4 ± 0.1	4.5 ± 0.1	0.42	0.90
FMD (%)	4.7 ± 0.8	3.7 ± 0.8	0.28		6.2 ± 0.9	5.5 ± 0.8	0.43	6.0 ± 0.8	5.5 ± 0.8	0.56	0.92
Peak shear rate (s^−1^)	277 ± 27	282 ± 31	0.88		323 ± 28	345 ± 33	0.59	336 ± 25	313 ± 30	0.53	0.70
Mean blood velocity (cm/s)	29.1 ± 2.4	29.6 ± 2.6	0.89		32.1 ± 2.5	34.1 ± 2.7	0.60	35.4 ± 2.2	34.0 ± 2.4	0.69	0.80

Note

Data represent mean ± SE.

Abbreviations: HIIT, High‐intensity interval training; Inter *p*, Interaction *p*‐value; MICT, Moderate‐intensity continuous training.

* denotes significant difference *p* < 0.05.

### 3.5 Exercise training and cardiorespiratory fitness

The mean total exercise time for each training session (including 10 min for warmup and cool down) was higher with MICT (37 ± 5 min) compared to HIIT (28 ± 2 min). Subjects in the HIIT group performed 10 ± 1 high‐intensity intervals per session (range: 8 to 12 intervals).

**FIGURE 3:**
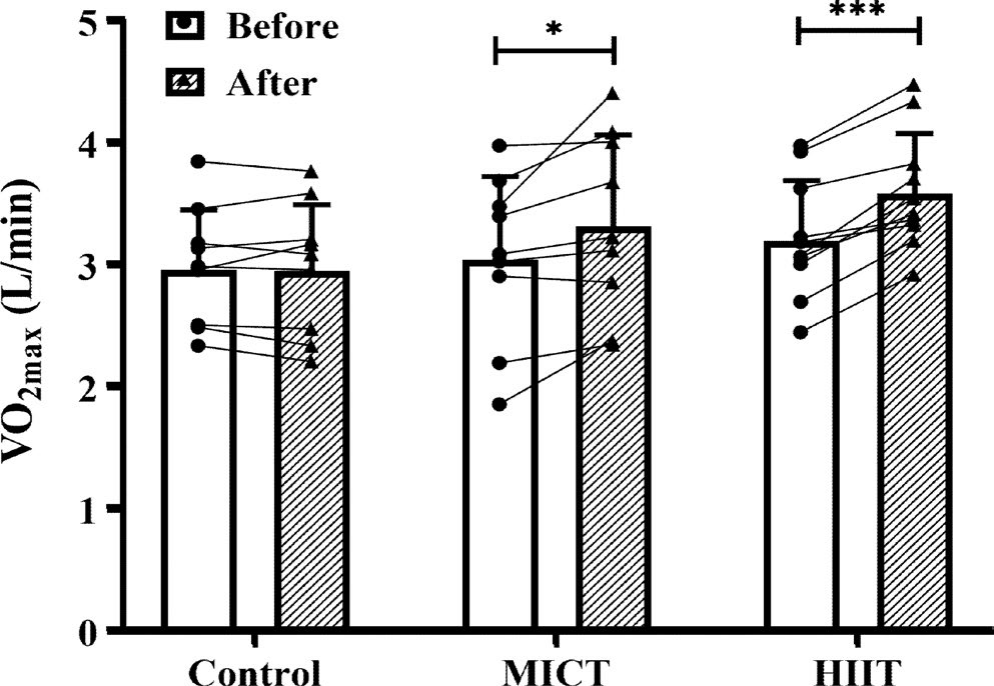
Maximal oxygen uptake (VO_2max_; L/min) before (open bars) and after (shaded bars) the 4‐week snacking intervention for control, moderate‐intensity continuous training (MICT), and high‐intensity interval training (HIIT) groups. Filled symbols represent individual data. Group data (bars) presented as means ± SD. *** *p* < 0.001 before vs. after within group. *p = 0.001 before vs. after within group

There was a time x group interaction for VO_2max_ (*p* = 0.001), with similar increases in MICT (9.2%; 3.05 ± 0.68 l/min to 3.33 ± 0.75 l/min; *p* = 0.001) and HIIT (12.1%; 3.21 ± 0.49 l/min to 3.60 ± 0.49 l/min; *p* < 0.001) but no change in control (2.97 ± 0.49 l/min to 2.96 ± 0.54 l/min; *p* = 0.88) (Figure 3).

## 4 DISCUSSION

We hypothesized that consumption of four dozen donuts as indulgent food snacks over 4 weeks would increase body weight and body fat, and that exercise training would attenuate the expected increase in body energy content. Surprisingly, consumption of ~14,500 kcal of this indulgent snack food consisting of 70% fat and sugar, without exercise training, resulted in no changes in mean body weight or body fat. Thus, we were unable to reject the null hypothesis for our primary outcome.

Previous studies on snacking demonstrate that some degree of energy compensation occurs, in which measured increases in body weight are invariably less than expected based on the additional energy content of the snacks (Claesson et al., 2009; Kant & Graubard, 2019; Lawton et al., 1998; Mazlan et al., 2006; Njike et al., 2017; Tey et al., 2011; Zaveri & Drummond, 2009). However, based on previous studies of snacking on indulgent foods, we anticipated that our control group would have gained body weight or body fat (Mazlan et al., 2006; Njike et al., 2017). The energy content of the 2‐donut snacks (6 days/week) in our study was ~600 kcal, which is similar to the energy content of the indulgent food snacks in previous studies that reported increases in body weight (Mazlan et al., 2006; Njike et al., 2017) or body fat (Njike et al., 2017).

We assessed dietary intake and physical activity levels at baseline and during the fourth week of the study. Results from our control group suggest that near‐complete compensation occurred for habitual energy and carbohydrate intake. However, even if we assume that the ~239 kcal/day lower energy intake during the fourth week is accurate, and we apply that to all 28 days of the 4‐week intervention, there is still a discrepancy of ~7,800 kcal between the energy content of the 48 donuts and the estimated reduction in energy intake.

Although our mean data suggest that energy compensation was essentially complete in the control group, there was considerable inter‐subject variability in changes in weight, body fat, fat‐free mass, and body energy content across all groups. Weight change ranged from a 1.8‐kg loss to a 3.1‐kg gain, and the change in energy content ranged from a decrease of 19,884 kcal to a gain of 17,241 kcal. Similar extreme changes in body weight (−2.3 kg to +6.3 kg) were reported in an observational study during a 5‐week winter holiday period (Stevenson et al., 2013). Even over longer periods of caloric restriction, overfeeding, or exercise training, large inter‐individual variability in body composition changes is well documented (Bouchard et al., 1990; Donnelly et al., 2003; King et al., 2008; Sawyer et al., 2015a). Our results demonstrate that large inter‐individual variability is also evident in response to snacking on indulgent foods such as donuts.

In the exercise training groups, the total energy cost of exercise training was ~4,000 kcal. Therefore, the exercise groups were expected to have an energy surplus of ~10,500 kcal if habitual dietary intake and physical activity levels did not change during the study. Dietary records indicated no change in energy intake, and accelerometer data indicated that neither group changed habitual physical activity during the intervention, as there were no significant changes in daily steps, sedentary time, or moderate‐to‐vigorous physical activity minutes. Our results are similar to those reported previously in studies of excess energy intake in the face of altered physical activity. Men who consumed an excess of 11,327 kcal over 7 days increased body energy content (DXA) by only 3,814 kcal and 6,824 kcal in control and exercise groups, respectively (Walhin et al., 2013). Similarly, in another study, an excess energy intake of ~29,000 kcal over 14 days resulted in an increase in body energy content of only 13,136 kcal (Krogh‐Madsen et al., 2014).

One previous study reported that 12 weeks of snacking on cookies increased “visceral fat rating” assessed by Tanita SC‐240 Body Composition Analyzer (Njike et al., 2017). Visceral abdominal fat was not increased in our control group, which may be due to a shorter duration of intervention. It could also be due to greater energy compensation or more precise assessment of visceral body fat (DXA). An unexpected finding of the present study was the 6% increase in visceral fat in the HIIT group. A high level of daily physical activity (~11,000 steps/day) has been shown to prevent increases in visceral abdominal fat during a 14‐day period of overfeeding (Krogh‐Madsen et al., 2014). The modest increase in visceral abdominal fat in HIIT compared to no change in the control group was unexpected, and we have no explanation for this finding. HIIT in the absence of dietary changes has been reported to reduce visceral abdominal fat, but a meta‐analysis indicated that lower intensity HIIT (<90 HR_max_) was more effective than higher intensity HIIT (>90 HR_max_) (Maillard et al., 2018). Our higher intensity HIIT program (90–95% HR_max_) may not have been optimal to prevent the 6% increase in visceral abdominal fat during the 4 weeks of snacking on donuts. Whether the modest increase in visceral abdominal fat in HIIT poses a health risk is of concern. It has previously been documented that visceral adipose tissue is associated with an increased risk of hypertension, dyslipidemia, and diabetes (Jensen, 1997; Piche et al., 2018). Furthermore, increases in visceral and abdominal adiposity during overfeeding have been shown to be strongly correlated with increased blood pressure and arterial stiffness in as little as 6–8 weeks (Fabbrini et al., 2015; Orr et al., 2008) and insulin resistance (Fabbrini et al., 2015; Knudsen et al., 2012; Krogh‐Madsen et al., 2014). Accordingly, it is important to note that blood pressure, blood lipids, and endothelial function were not adversely affected in the HIIT group in the current study.

We did not observe any deleterious changes in blood pressure, blood lipids, or insulin resistance indices across all groups. In a prior study, snacking on cookies over 12 weeks in overweight and obese middle‐aged men and women (mean age = 56.5 yr; BMI = 34.4 kg/m^2^) increased systolic and diastolic blood pressures by 6.2 mmHg and 3.9 mmHg, respectively (Njike et al., 2017). Mean resting blood pressures in that study were 121.4/71.6 mmHg, slightly lower than blood pressures in our subjects. The lack of increase in blood pressures in our subjects may be due to the shorter duration of our study (4 weeks vs. 12 weeks). Furthermore, the increase in blood pressures in the previous study may also be due in part to the increase in both body fat percentage (+6.2%, from a baseline of 38.3%) and visceral fat rating (Njike et al., 2017). In contrast, total body fat was unchanged in our subjects, and visceral abdominal fat was unchanged in our control and MICT groups. Any potential adverse effect on blood pressure from the visceral abdominal fat increase in HIIT could have been offset by the exercise training. Finally, the lack of change in blood lipids observed in our study is consistent with results from other studies of indulgent food snacking (Claesson et al., 2009; Njike et al., 2017; Tey et al., 2011), even with excess energy intake from candy of ~1360 kcal/day (Claesson et al., 2009).

Studies of the effects of indulgent food snacking on glucose tolerance and insulin action are limited. In young men and women (mean BMI = 22.2 kg/m^2^), 2 weeks of compulsory consumption of 20 kcal/kg/day (~1,360 kcal) of candy, while instructed to maintain a normal diet, increased fasting insulin concentration by 44% (Claesson et al., 2009). However, glucose tolerance was not assessed in that study, nor in other studies of snacking on indulgent foods (Lawton et al., 1998; Mazlan et al., 2006; Njike et al., 2017; Tey et al., 2011). In overfeeding studies with (Krogh‐Madsen et al., 2014; Walhin et al., 2013) or without (Kechagias et al., 2008) mandatory decreases in daily step counts over 7–14 days, deterioration of glucose tolerance and increases in fasting and 2‐hour insulin concentrations have been reported. Unexpectedly, glucose tolerance improved in our control subjects as evidenced by a ~26% reduction in glucose iAUC during the OGTT. This improvement was comparable to the ~33% reduction in glucose iAUC in MICT. The improved glucose tolerance in MICT might be expected given the beneficial effects of exercise training on glucose metabolism. However, the lack of improvement in glucose tolerance in HIIT is difficult to explain (Angelopoulos et al., 2002; Babraj et al., 2009), especially since HIIT and MICT programs similar to those used in the present study have been reported to increase insulin sensitivity (Ryan et al., 2020) and improve glucose tolerance (Malin et al., 2019) by the same amount in overweight and obese men. However, it is important to note that this was a secondary outcome, and the study was not powered to detect differences in glucose tolerance. Even more puzzling is the improvement in glucose tolerance in the control group. To our knowledge, this is the first time this has been reported. Additional research is necessary to clarify whether this is a spurious finding.

Endothelial function, assessed by FMD, was unchanged in all groups. We previously demonstrated that 4 weeks of HIIT in obese men and women increased FMD by 51% (4.82% to 7.29%) (Sawyer et al., 2016). Training frequency in that study was 3 days/week, compared to 4 days/week in the present study. Thus, the training dose was slightly greater in the present study, which makes the lack of increase in FMD even more surprising. Although the consumption of ~14,500 kcal of donuts was not sufficient to impair FMD in our sedentary control group, it may have prevented the expected increase in FMD in our HIIT group. Because our previous study (Sawyer et al., 2016) indicated that a similar MICT protocol did not increase FMD after 4 weeks or 8 weeks of training in obese men and women, the lack of increase in FMD in the current MICT group was not surprising.

Although none of the previous studies of snacking assessed FMD, a recent study by Bock et al. (Bock et al., 2020) demonstrated that 1 week of consuming 900 kcal/day of sugar‐sweetened beverages (SSBs), while maintaining a normal diet, impaired FMD in young, normal weight men. In subjects randomized to exercise training, the addition of 45 min of moderate‐intensity cycling (60–65% of age‐predicted HR_max_) on 5 of the 7 intervention days (900 kcal/day of SSBs) not only prevented an impairment in endothelial function, but improved FMD by 18%. In our study, the energy content of the two donuts consumed each day (600 kcal) was less than the energy content of the SSBs (900 kcal) consumed in the study by Bock et al. (2020). As such, it is possible that our snack “dose” was not high enough in kcal and sugar to induce adverse cardiometabolic effects.

### 4.1 Strengths and limitations

Our study has several strengths. This is the only study to examine the effects of indulgent food snacking, with or without exercise training, on men with BMI ≥25 kg/m^2^. Our use of donuts as indulgent snack is novel and has ecological value since donuts are widely consumed and consist mainly of fat and sugar. Also, the additional energy content of the snacks (~600 kcal/day) is more likely to represent real‐life situations in comparison to the short‐term overfeeding studies in which energy excess was >1,500 kcal coupled with decreases in physical activity (Knudsen et al., 2012; Krogh‐Madsen et al., 2014; Walhin et al., 2013). We used DXA (considered the practical gold standard) to measure changes in body composition. Finally, all exercise training sessions were supervised with 100% adherence to prescribed exercise intensity and volume.

This study is not without limitations. The study duration was relatively short. However, increases in body weight have been reported in snacking studies as short as 1–2 weeks (Claesson et al., 2009; Mazlan et al., 2006). Reliance on self‐report dietary intake is a limitation as it has been shown to be prone to underreporting, especially in overweight and obese individuals (Dhurandhar et al., 2015; Lichtman et al., 1992; Schoeller et al., 1990). However, an in‐patient study with strict dietary control (all meals fed to participants) was not feasible at our institution. Nonetheless, the self‐report dietary data in the control group suggest that energy intake compensation occurred, which is consistent with previous studies on snacking (Claesson et al., 2009; Kant & Graubard, 2019; Lawton et al., 1998; Mazlan et al., 2006; Njike et al., 2017; Tey et al., 2011; Zaveri & Drummond, 2009). We did not have a gold‐standard assessment of total energy expenditure such as doubly labeled water. Thus, we cannot be sure that total daily energy expenditure was not affected by donut consumption or exercise training. Increases in nonexercise activity thermogenesis (NEAT) may have occurred which could have offset the additional energy consumed from the donuts (Levine, 2007). Fat gain during an 8‐week period of overfeeding was reported to be inversely correlated with the magnitude of NEAT in nonobese adults (Levine et al., 1999). We did not measure resting metabolic rate, which might have changed during the study. However, it is not likely that resting metabolic rate would have changed significantly in our subjects. Increases in resting metabolic rate during overfeeding is a function of changes in fat‐free mass (Westerterp, 2017). Also, 14 days of consuming an additional 20 kcal/kg body weight per day (~1360 kcal/day) as candy did not increase resting metabolic rate in nonobese adults (Claesson et al., 2009). Finally, a recent meta‐analysis reported that aerobic exercise training has no appreciable effect on resting metabolic rate (MacKenzie‐Shalders et al., 2020). During the 4‐week intervention, accelerometry data were only collected during the fourth week, so it is possible that the measurements during week 4 did not accurately reflect physical activity during the entire 4 weeks of snacking. However, the lack of increase in VO_2max_ in the control group suggests that control subjects did not perform any additional unmeasured physical activity during the intervention period sufficient to improve cardiorespiratory fitness.

## 5 CONCLUSION

Consuming ~14,500 additional kcal (48 donuts) over 4 weeks did not result in a gain in body weight or body fat and did not cause deterioration in several markers of cardiometabolic risk in overweight and obese men. In fact, oral glucose tolerance was improved in the control group despite no change in objectively measured physical activity during the 4‐week period. Energy intake compensation likely occurred, as diet records indicated a reduction in total daily energy and carbohydrate intake. Both HIIT and MICT improved cardiorespiratory fitness but, aside from a reduction in glucose iAUC in MICT, experienced no improvements in blood pressure, blood lipids, or endothelial function. The lack of improvement in FMD following HIIT suggests that consumption of the donuts may have prevented the expected exercise training‐induced improvement in endothelial function.

## DISCLOSURES

No conflicts of interest, financial or otherwise, are declared by the authors.

## AUTHOR CONTRIBUTIONS

W.J.T., S.S.A., and G.A.G. conception and study design. W.J.T., C.L.J., and A.C.D. performed experiments and collected data. W.J.T. analyzed data. W.J.T., C.L.J., A.C.D., S.S.A., and G.A.G. interpreted results of experiments; W.J.T. prepared tables and figures; W.J.T. and G.A.G. drafted the manuscript; W.J.T., C.L.J., A.C.D., S.S.A., and G.A.G. edited and revised the manuscript; W.J.T., C.L.J., A.C.D., S.S.A., and G.A.G. approved the final version of the manuscript.
